# Marine *Synechococcus* sp. Strain WH7803 Shows Specific Adaptative Responses to Assimilate Nanomolar Concentrations of Nitrate

**DOI:** 10.1128/spectrum.00187-22

**Published:** 2022-07-19

**Authors:** María Agustina Domínguez-Martín, Antonio López-Lozano, Yesica Melero-Rubio, Guadalupe Gómez-Baena, Juan Andrés Jiménez-Estrada, Kateryna Kukil, Jesús Diez, José Manuel García-Fernández

**Affiliations:** a Departamento de Bioquímica y Biología Molecular, Campus de Excelencia Internacional Agroalimentario CeiA3, Universidad de Córdoba, Córdoba, Spain; University of Mississippi

**Keywords:** *Synechococcus* WH7803, nitrate, nitrogen metabolism, proteomics, transcriptomics

## Abstract

Marine *Synechococcus*, together with *Prochlorococcus*, contribute to a significant proportion of the primary production on Earth. The spatial distribution of these two groups of marine picocyanobacteria depends on different factors such as nutrient availability and temperature. Some *Synechococcus* ecotypes thrive in mesotrophic and moderately oligotrophic waters, where they exploit both oxidized and reduced forms of nitrogen. Here, we present a comprehensive study, which includes transcriptomic and proteomic analyses of the response of *Synechococcus* sp. strain WH7803 to nanomolar concentrations of nitrate, compared to micromolar ammonium or nitrogen starvation. We found that *Synechococcus* has a specific response to a nanomolar nitrate concentration that differs from the response shown under nitrogen starvation or the presence of standard concentrations of either ammonium or nitrate. This fact suggests that the particular response to the uptake of nanomolar concentrations of nitrate could be an evolutionary advantage for marine *Synechococcus* against *Prochlorococcus* in the natural environment.

**IMPORTANCE** Marine *Synechococcus* are a very abundant group of photosynthetic organisms on our planet. Previous studies have shown blooms of these organisms when nanomolar concentrations of nitrate become available. We have assessed the effect of nanomolar nitrate concentrations by studying the transcriptome and proteome of *Synechococcus* sp. WH7803, together with some physiological parameters. We found evidence that *Synechococcus* sp. strain WH7803 does sense and react to nanomolar concentrations of nitrate, suggesting the occurrence of specific adaptive mechanisms to allow their utilization. Thus, very low concentrations of nitrate in the ocean seem to be a significant nitrogen source for marine picocyanobacteria.

## INTRODUCTION

Nitrogen is generally accepted as one of the most common nutrients that limit phytoplankton primary production in marine ecosystems (1). The largest reservoir of nitrogen in the sea is in the form of dissolved dinitrogen gas (N_2_), although it can also be found as nitrate, nitrite, and ammonium at the nanomolar range in surface waters (2). Unicellular cyanobacteria of the genera *Synechococcus* and *Prochlorococcus* contribute to a significant proportion of phytoplankton biomass production in the ocean (3–5). The spatial distribution of both groups of marine picocyanobacteria is influenced by a variety of factors, including nutrient availability and temperature (3, 6–10). While *Prochlorococcus* is abundant in the nutrient-poor waters of subtropical and tropical areas, *Synechococcus* thrives in mesotrophic and moderately oligotrophic waters, colonizing a wider range of ecological niches (4, 11). *Synechococcus* sp. strain WH7803 (here referred to as *Synechococcus* WH7803) was isolated in mesotrophic waters (12) and is the best-physiologically characterized marine *Synechococcus* strain.

Ammonium is the preferred nitrogen source for cyanobacteria, and therefore, it would be consumed first if provided together with other suitable nitrogen-containing compounds (13). Central to nitrogen metabolism is the assimilation of ammonium into organic molecules through the glutamine synthetase-glutamate synthase (GS-GOGAT) cycle, appearing as the connecting point between carbon and nitrogen metabolism. The assimilation of other inorganic forms of nitrogen requires additional energy and reducing power compared to that required to assimilate ammonium since they need to be transformed into ammonium to be eventually assimilated (14).

To maintain their metabolic homeostasis, bacteria must coordinate the flow rates of carbon and nitrogen assimilation. In cyanobacteria, the ammonium-promoted repression of nitrate or dinitrogen assimilation is controlled by 2-oxoglutarate (2-OG) intracellular levels (15–21). Due to the absence of the enzyme 2-OG dehydrogenase in marine *Synechococcus* and *Prochlorococcus*, 2-OG only serves as the acceptor for the newly assimilated nitrogen to form glutamate, and its concentration will change according to the nitrogen status of the cell (22). Thus, 2-OG acts as signaling molecule in a regulatory system including the transcription factor NtcA, the sensor-transducer protein P_II_, and the signaling protein PipX (23). NtcA is a ubiquitous protein in cyanobacteria which regulates transcription of genes related to nitrogen metabolism acting either as an activator or as a repressor (24, 25). The canonical NtcA-activated promoter is composed of a palindromic sequence GTAN_8_TAC centered around −40.5 with respect to the transcription start point and a −10 Pribnow-like box with the consensus sequence TAN_3_T (25, 26).

Most cyanobacteria have the capability to take up and assimilate nitrate (14, 22, 27). Both nitrate uptake and reduction are driven in cyanobacteria by photosynthetically generated assimilatory power (ATP and reduced ferredoxin). The reduction of nitrate to ammonium is catalyzed, in two successive steps, by nitrate reductase and nitrite reductase, both ferredoxin-dependent enzymes in cyanobacteria (28).

For the specific uptake of nitrate, two structurally distinct transporters have been identified in cyanobacteria, seemingly determined by the ecosystems where they thrive: an ABC-type nitrate/nitrite transporter (ABC-NRT) encoded by the *nrtA*, *nrtB*, *nrtC*, and *nrtD* genes (29, 30) for most freshwater cyanobacteria and a high-affinity permease from the major facilitator superfamily (MFS) encoded by the *nrtP* gene (also designated *napA*) (31, 32) for marine cyanobacteria. While the ABC-NRT shows high affinity for both nitrate and nitrite (with a *K_m_* value of about 1 μM), the affinity for nitrite is much lower than that for nitrate in the case of the NrtP transporter (33). Related to this feature shown by NrtP, most marine cyanobacteria (but rarely freshwater strains) have a nitrite transporter (NitM) from the formate/nitrite transporter (FNT) family (also designated FocA), encoded by a gene tightly linked with that encoding nitrite reductase, allowing a high-affinity uptake of nitrite in those strains with NrtP instead of ABC-NRT (34).

Moreover, the versatility of marine *Synechococcus* strains has been related to its ability to grow over a wide range of light intensities and spectral qualities (35) and to utilize a wide variety of nitrogen sources. Different isolates of marine *Synechococcus* have been reported to utilize ammonium, nitrate, nitrite, urea, and amino acids as the sole nitrogen source while employing systems for active uptake of these compounds (36–39).

With respect to *Synechococcus* WH7803, it has been described that in the stratified Sargasso seawater (where this strain was isolated), nanomolar changes in nitrate concentrations occurred. This change was stoichiometrically consistent with the subsequent cellular production of a bloom of these cyanobacteria (40).

The question that arises is how marine *Synechococcus* are able to successfully coexist with *Prochlorococcus* spp. even though they are apparently inferior in their capacity to take low concentrations of reduced forms of nitrogen such as ammonium (41). One possible answer is that *Synechococcus* may be more efficient at utilizing low concentrations of nitrate, which is generally less available to *Prochlorococcus* (since only some strains of this genus are enabled to utilize nitrate [42]). In this work, we demonstrate through different approaches that *Synechococcus* effectively shows a differential response when a nanomolar nitrate concentration is available.

## RESULTS

### Genomic context and NtcA-regulated promoters.

**FIG 1:**
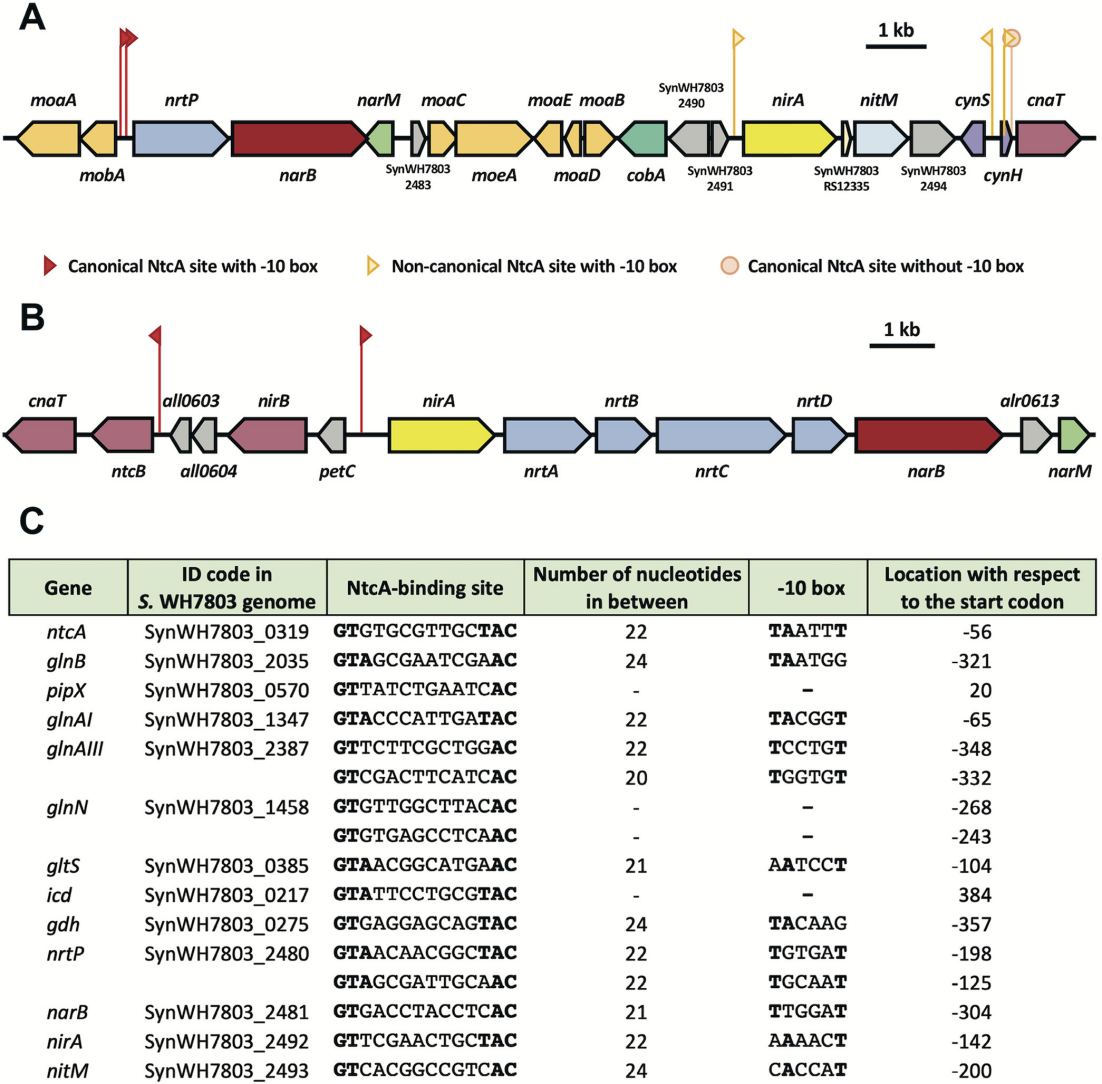
Organization of the nitrate assimilation genes. (A) Nitrate assimilation gene cluster in *Synechococcus* WH7803. (B) Nitrate assimilation gene cluster in *Nostoc* sp. strain PCC 7120. (C) Predicted NtcA-regulated promoters in *Synechococcus* sp. WH7803. The predicted target regions for genes involved in nitrogen metabolism in *Synechococcus* sp. WH7803 are listed. The location of the NtcA-binding site with respect to the start codon of the corresponding gene is indicated. Nucleotides matching those of the consensus NtcA-binding site (GTAN_8_TAC) and of the −10 box (TAN_3_T) are in boldface.

The nitrate assimilation gene cluster in the genome of *Synechococcus* WH7803 encompasses genes encoding all needed molecular elements to take up nitrate and/or nitrite into the cell and to reduce these inorganic nitrogen compounds to ammonium. Figure 1A shows a scheme of the *Synechococcus* WH7803 genomic region bearing its nitrate assimilation gene cluster distributed along 13,300 bp. Accordingly with the most common arrangement of the nitrate assimilation genes in marine cyanobacteria, *nrtP* and *narB* genes (encoding the NrtP transporter and nitrate reductase, respectively), are closely located and transcribed in the same orientation, with only 82 bp between the end of the *nrtP* coding sequence and the start codon of *narB*. Although *nirA*, encoding nitrite reductase, frequently takes part of the same operon together with genes encoding the nitrate/nitrite transporter and nitrate reductase in freshwater cyanobacteria (Fig. 1B), that is not the case for marine cyanobacteria (22). In *Synechococcus* WH7803, *nirA* is separated from *nrtP* and *narB* by exactly 6,000 bp, taking part in its own putative operon together with the nitrite transporter gene *nitM*.

Although the two types of active transporters for nitrate, the ABC-type encoded by *nrtABCD* and the MFS transporter encoded by the *nrtP* gene, are differentially distributed among the cyanobacterial groups, there are some exceptions. Nostoc punctiforme ATCC 29133 is an example of a freshwater cyanobacterial strain that has NrtP as the only nitrate transporter, while *Synechococcus* sp. WH5101 is a marine cyanobacterium, with the ABC-type transporter as the sole transporter for nitrate (43). However, there are also known examples of freshwater cyanobacterial strains including the *nrtP* gene in their genomes, coexisting with the *nrtABCD* cluster encoding the ABC-type nitrate transporter (22). These cyanobacteria, i.e., Arthrospira polantensis NIES-39 and *Cyanothece* sp. strains PCC 7424, PCC 8801, and PCC 8802, may get enhanced capacity for adaptation to changing environmental conditions by having both NrtP and the ABC-type transporters (22).

The repression of nitrate assimilation genes by the presence of ammonium in cyanobacteria is mediated by NtcA, and it is not affected by the presence or absence of nitrate (27). Thus, an increase in the intracellular 2-OG levels leads to the activation of the so-called global nitrogen regulator NtcA, enhancing the expression of most genes involved in nitrate assimilation in cyanobacteria. Two putative NtcA-regulated promoters were identified 125 and 198 bp upstream of the *nrtP* translational start site (tss) with the structure GTAGCGATTGCAAC-N_22_-TGCAAT (with TAC changed to AAC and TAN_3_T changed to TGN_3_T) and GTAACAACGGCTAC-N_22_-TGTGAT (with TAN_3_T changed to TGN_3_T), respectively, while a less canonical putative site was found 304 bp upstream of the *narB* tss, centered 221 bp upstream of the stop codon of *nrtP*: GTGACCTACCTCAC-N_21_-TTGGAT (with GTA and TAC changed to GTG and CAC, respectively, and TAN_3_T changed to TTN_3_T). For *nirA*, a putative NtcA-binding site was identified 142 bp upstream of this gene tss with the sequence GTTCGAACTGCTAC-N_22_-AAAACT (with GTA changed to GTT and TAN_3_T changed to AAN_3_T), while another putative site was found 200 bp upstream of the *nitM* tss: GTCACGGCCGTCAC-N_24_-CACCAT (with GTA and TAC changed to GTC and CAC, respectively, and TAN_3_T changed to CAN_3_T).

In the *Synechococcus* WH7803 genomic region bearing this nitrate assimilation gene cluster, two additional genes associated with an NtcA-binding site can be identified: *cynS*, encoding cyanase, the enzyme catalyzing the bicarbonate-dependent decomposition of cyanate into ammonium and CO_2_, and *cnaT*, encoding a positive regulatory element of the *nirA* operon expression as described for *Nostoc* sp. strain PCC 7120 (44). A putative NtcA-regulated promoter was identified 127 bp upstream of the *cynS* tss with the structure GTGACTGCGTGAAC-N_23_-TGGCAT (with GTA and TAC changed to GTG and AAC, respectively, and TAN_3_T changed to TGN_3_T), while an NtcA-binding site linked to a −10 box was found for the *cnaT* gene: GTTCAGCCAGCAAC-N_22_-TCTGGT (with GTA and TAC changed to GTT and AAC, respectively, and TAN_3_T changed to TCN_3_T), centered 213 bp upstream of this gene tss. Additionally, a canonical NtcA-binding site, GTATCAACGACTAC, is placed 57 bp upstream of the *cnaT* tss, although no recognizable –10 box is associated with it.

The sequences for the predicted NtcA-binding sites linked to genes related to the nitrate assimilation gene cluster, together with those predicted for genes involved in the assimilation of other nitrogen sources as well as its regulation in *Synechococcus* WH7803 (experimentally determined only for *ntcA*), were used to define the likely structure of the consensus NtcA-binding site for this strain (Fig. 1C).

### Effect of different nitrogen sources on the *Synechococcus* WH7803 metabolism.

Different parameters indicating the status of *Synechococcus* WH7803 cultures were measured to determine the effect of various nitrogen availabilities. Cultures were subjected to four experimental conditions: nitrogen starvation, addition of 800 μM ammonium, and addition of two nitrate concentrations, 800 nM and 800 μM. We used nonaxenic cultures, which could be more representative of the natural physiological conditions for *Synechococcus* WH7803, since marine picocyanobacteria have been shown to rely on interactions with heterotrophic bacteria for key aspects of their metabolism (45, 46).

**FIG 2:**
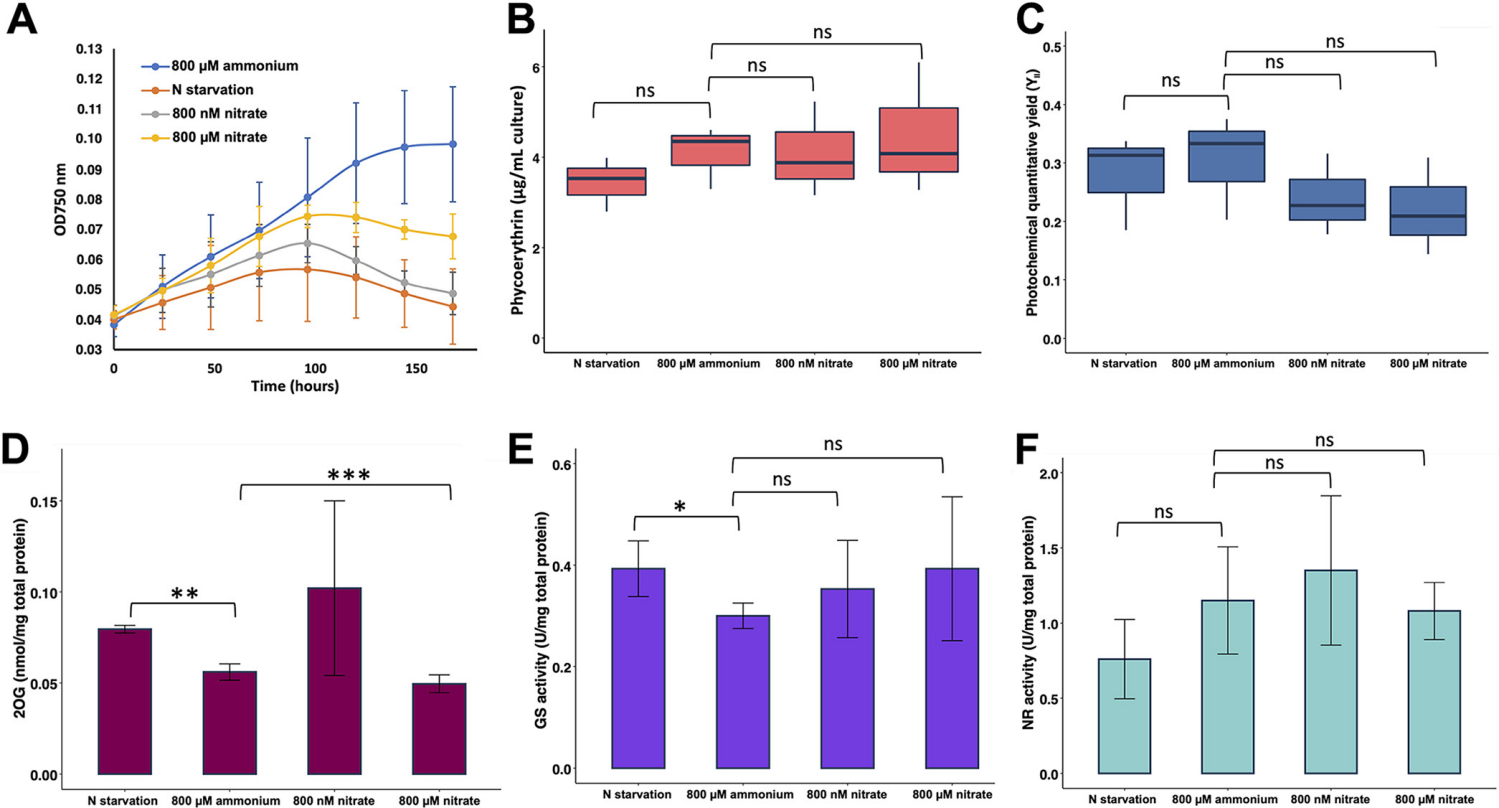
Physiological parameters and enzymatic activities from *Synechococcus* WH7803 cultures under different nitrogen sources after 24 h. (A) Growth curve. (B) Phycoerythrin (PE) content. (C) Photochemical quantum yield of PSII (Y_II_). (D) Intracellular concentration of 2-OG. (E) GS transferase activity. (F) Nitrate reductase activity. ns, not statistically significant; *, *P* < 0.05; **, *P* < 0.01. The error bars represent the standard deviation. The results are from at least three independent biological replicates.

Traditionally, the freshwater cyanobacteria have been grown in micromolar concentrations of nitrate (47). In our experimental setup, *Synechococcus* WH7803 cells grew normally under both concentrations of nitrate, although the micromolar concentration of nitrate showed a higher growth rate than the nanomolar condition (Fig. 2A). As was expected for a non-nitrogen-fixing cyanobacterium, the lack of nitrogen negatively affected the *Synechococcus* WH7803 growth, while higher rates were observed in cultures with ammonium as the sole nitrogen source (Fig. 2A).

Since the removal of nitrogen sources from growth medium generally causes chlorosis in cyanobacteria (48, 49), the effect of nitrogen starvation and nanomolar concentrations of nitrate on the content of phycoerythrin (PE) was determined (Fig. 2B). The results showed a reduction close to 16% in the PE content after 24 h of nitrogen starvation. However, PE content in cultures supplemented with different values of nitrate concentrations did not significantly change.

Another important parameter to monitor the status of cells in a cyanobacterial culture is its effective photochemical quantum yield of photosystem II (Y_II_). The effect of different nitrogen concentrations on *Synechococcus* WH7803 Y_II_ is shown in Fig. 2C. The results did not show statistically significant (*t* test) differences among the tested sources after 24 h; therefore, the data obtained suggest that photosynthesis is not strongly affected.

In cyanobacteria, the signal controlling the C/N balance is the level of intracellular 2-OG (13, 20, 26, 28, 50). The concentration of intracellular 2-OG was determined when *Synechococcus* WH7803 cells were grown under different nitrogen sources (Fig. 2D). With ammonium at 800 μM, the 2-OG concentration was about 0.05 nmol/mg of protein, a concentration similar to that observed in cultures growing with 800 μM nitrate for 24 h. Although the level of 2-OG slightly increased under nanomolar nitrate concentrations with respect to 800 μM ammonium or nitrate, it only reached a significant value under nitrogen starvation (*P* = 0.0011), suggesting that the nanomolar nitrate condition may be an intermediate situation, in which the machinery to assimilate nitrate is still upregulated.

Glutamine synthetase (GS, EC 6.3.1.2) and nitrate reductase (NR, EC 1.7.7.2) are two key enzymes in nitrogen metabolism. Nitrate reductase catalyzes the first reaction in the two-step reduction of nitrate to ammonium, while glutamine synthetase is responsible for the incorporation of ammonium into organic N compounds. Both activities were determined under different nitrogen sources in *Synechococcus* WH7803 (Fig. 2E and F). The GS transferase activity increased under nitrogen starvation (*P* = 0.0494) compared to that detected for cells growing with ammonium as the nitrogen source. Nevertheless, cultures under nitrate (at nanomolar and micromolar concentrations) did not change significantly compared with the control condition under ammonium (Fig. 2E). Regarding the NR activity, there are appreciable differences among different conditions, but they are not statistically significant. It is worth pointing out that the cells were routinely grown on ammonium as the sole N source, and when ammonium was present, there was nitrate reductase activity.

### Transcriptomic analysis: nitrogen starvation versus nanomolar nitrate concentration in *Synechococcus* WH7803.

Transcriptomic analyses were carried out to get a comprehensive overview of the response to the lack of nitrogen versus the addition of a nanomolar concentration of nitrate.

**FIG 3:**
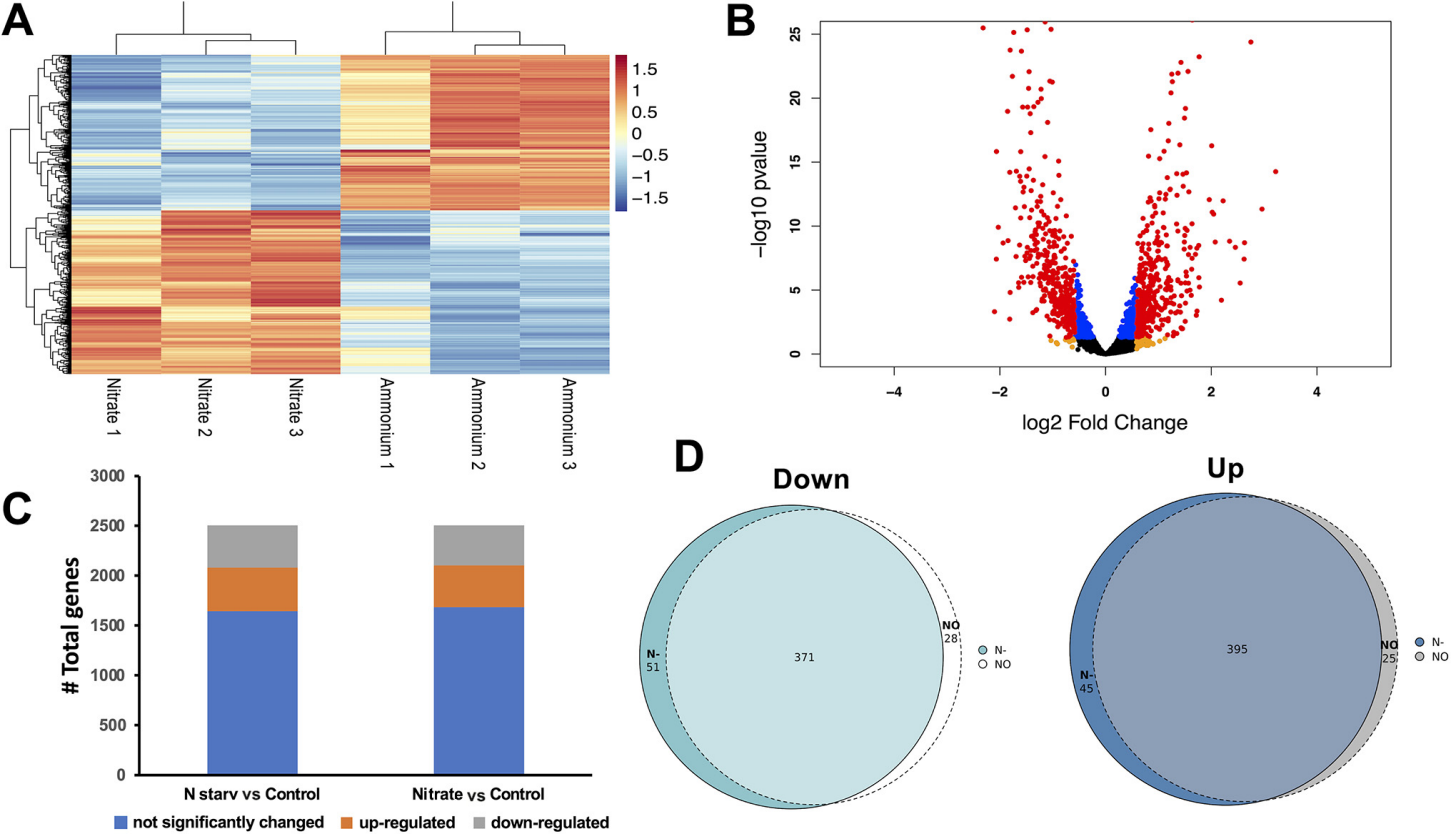
Number of differentially expressed genes in *Synechococcus* WH7803 under the different conditions tested. (A) Heatmap, 800 μM ammonium (ammonium) and 800 nM nitrate (nitrate). 1, 2, and 3 represent biological replicates. (B) Volcano plot zoom –log_10_ *P* value adjustment of 0.25. (C) Bar plot showing the changed genes in the two comparisons: nitrogen starvation (N starv) versus 800 μM ammonium (ammonium) and 800 nM nitrate (Nitrate) versus 800 μM ammonium (ammonium). (D) Venn diagram of the downregulated (down) and upregulated (up) genes: nitrogen starvation (N–) versus 800 nM nitrate (NO).

The genome of *Synechococcus* WH7803 encodes 2,530 proteins (UP000001566 in the UniProt database). This transcriptomic analysis identified 2,505 genes representing 99% of the genome (see Table S1 in the supplemental material). Using a cutoff of a >1.5-fold change and a *P* adjustment value of <0.05, 862 and 819 genes showed differential expression under nitrogen starvation or with a nanomolar concentration of nitrate, respectively, compared to ammonium (Fig. 3A, Fig. S1B and Tables S2 and S3).

The volcano plot highlights the proportion of the significant changes and the magnitude of those changes (Fig. 3B and Fig. S1A). From the upregulated genes (Fig. 3C), 395 were common to both conditions, 45 genes were specifically upregulated under nitrogen starvation, and 25 specifically were upregulated under a nanomolar concentration of nitrate (Fig. 3D). Moreover, 371 common genes were downregulated under the two conditions, 28 genes were specifically downregulated when the cells were grown under a nanomolar concentration of nitrate, and 51 were upregulated in response to the lack of nitrogen (Fig. 3D). At the transcriptomic level, more genes respond specifically to the nitrogen starvation condition.

Furthermore, global analysis of the differential expression using the portal DAVID (51) has been done (Fig. S2 and S3), highlighting the main transcriptional changes observed in our experiment.

Significant changes were observed for 168 upregulated and 134 downregulated open reading frames (ORFs) encoding gene products labeled “hypothetical proteins” by the NCBI database for the *Synechococcus* WH7803 genome (CT971583). Gene IDs for all these ORFs were used to retrieve the corresponding UniProt entries (https://www.uniprot.org/uploadlists), allowing the identification of 67 of the upregulated (39.88%) and 52 of the downregulated (38.81%) genes (Table S4).

Genes encoding the main enzymes and regulatory proteins involved in nitrogen metabolism which showed an increase in their expression with respect to the ammonium control did not, however, exhibit a differential response under both nitrogen starvation and nanomolar nitrate concentration conditions (here, values of fold change for nitrogen starvation versus nanomolar nitrate with respect to the ammonium control will be indicated for each mentioned gene). That was the case for *ntcA* (13.84 versus 13.28), *glnB* (2.06 versus 2.09), encoding the signal transduction protein P_II_, *glnA* (2.10 versus 2.01), encoding glutamine synthetase, *amt1* (45.95 versus 47.39), encoding the ammonium transporter, *cynS* (273.76 versus 289.39), encoding cyanase, *nrtP* (20.40 versus 19.12), *narB* (7.53 versus 7.56), *nirA* (159.15 versus 161.64), and *nitM* (55.87 versus 58.75). It is worth noting that the highest values of expression under both experimental conditions included those obtained for genes encoding the key proteins involved in nitrate assimilation: nitrate transporter NrtP, nitrate reductase, nitrite transporter NitM and nitrite reductase, although the expression of these genes did not seem to be affected by the addition of nanomolar concentrations of nitrate with respect to nitrogen starvation. The *cynS* gene, with a putative NtcA-regulated promoter, showed very high values of expression, even higher in cultures growing under nanomolar nitrate concentrations. However, other genes, such as *icd*, encoding isocitrate dehydrogenase (the enzyme catalyzing the production of 2-OG in the tricarboxylic acid cycle), or *pipX*, encoding the signaling protein PipX, which are generally upregulated by nitrogen starvation in cyanobacteria, did not show a significant variation of their expressions under these conditions.

An essential part of the ecological success of the marine picocyanobacteria is the nutrient acquisition mechanisms, such as the use of high-affinity ATP-binding cassette (ABC) transporters to competitively acquire nutrients, especially in oligotrophic marine environments (52). Our transcriptomic analysis identified many annotated ABC transporters and permeases (Tables S2 and S3). Most of the transporters were similarly downregulated under nitrogen starvation and with a nanomolar concentration of nitrate (Tables S2 and S3). Interestingly, several phosphate ABC transporters were downregulated (*SynWH7803_1243*; *SynWH7803_1470*; *SynWH7803_0137*; *SynWH7803_1244*; *SynWH7803_1045*; *SynWH7803_1471*, and *SynWH7803_1469*). Two transporters were downregulated only in the nanomolar nitrate cultures (*SynWH7803_1470* and *SynWH7803_0137*), and other one only under nitrogen starvation (*SynWH7803_1469*). Furthermore, under our study conditions, another set of transporters and permeases was upregulated. The ammonium transporter showed slightly lower expression on the cultures grown in the absence of nitrogen sources with respect to those at a nanomolar nitrate concentration (45.9 versus 47.4). Moreover, the nitrite transporter showed a similar pattern (55.8 versus 58.7). As we expected, the MFS nitrate transporter was upregulated, although the level of expression was almost the same at both conditions (20.4 versus 19.1).

Although the Y_II_ of *Synechococcus* WH7803 cultures growing in nitrogen starvation or at a nitrate nanomolar concentration seemed not to be affected compared to the control culture, the expression of most genes encoding subunits from photosystems I and II displayed a general downregulation as follows: from photosystem I, *psaB* (−2.46 versus −2.20), *psaI* (−2.00 versus −1.72), *psaK* (−2.77 versus −2.34), and *psaL* (−3.08 versus −2.65) and from photosystem II, *psbA* (−2.01 versus −1.72), *psbL* (−1.90 versus −1.57), and *psbM* (−2.50 versus −2.19). Additionally, there are two copies of the genes encoding the photosynthetic pigment PE, *cpeBA* and *mpeBA* (53), in the genome of *Synechococcus* WH7803. Both copies share the same genomic region with the *cpeCDESTR* operon, encoding linker polypeptides that associate with PE, an activator required for transcription of both operons (54), and other genes related to the synthesis of the phycobilisome (55). While this whole set of genes showed a general downregulation, some of them revealed the clearest differential response to the absence of nitrate versus the addition of a nanomolar concentration of nitrate: *cpeB* (−4.07 versus −3.50), *cpeA* (−5.69 versus −4.58), *mpeE* (−4.09 versus −3.52), *cpeE* (−3.55 versus −3.05), and *cpcG2* (−3.79 versus −3.08). The absence of nitrogen for PE synthesis seems to lead to the downregulation of the genes involved in this pigment synthesis, although the availability of just a low concentration of nitrate could be perceived by *Synechococcus* WH7803 cells, and consequently, the negative effect on the transcription of those genes would get reduced.

As might be expected for a photosynthetic organism, a general downregulation observed for genes encoding the subunits of the photosynthetic apparatus and those proteins involved in the synthesis of the phycobilisome was also observed for genes related to the energetic metabolism of *Synechococcus* WH7803. With no significant differences between nitrogen starvation and nanomolar nitrate concentration conditions, the expression of genes encoding the ATP synthase subunits decreased with respect to the control condition (800 μM ammonium): *atpE* (−3.49 versus −3.04), *atpC* (−5.47 versus −5.05), *atpA* (−6.27 versus −6.07), *atpD* (−7.19 versus −6.60), *atpF* (−5.70 versus −5.85), *atpG* (−6.52 versus −7.22), *atpH* (−8.72 versus −8.14), and *atpI* (−5.03 versus −4.98). Furthermore, genes encoding subunits of the ribulose 1,5-bisphosphate carboxylase (RuBisCO) together with those encoding carboxysome shell proteins were also downregulated under both conditions: *rbcL* (−3.95 versus −3.34), *rbcS* (−5.80 versus −4.45), *csoS2* (–6.13 versus −4.99), *csoS3* (−7.18 versus 6.18), *ccmL* (−2.56 versus −2.80), and *csoS4* (−3.77 versus −4.19). It is worth noting that most of these genes related to carbon fixation showed a differential response to the absence of nitrate versus the addition of a nanomolar concentration of nitrate, as was also the case for photosynthesis-related genes.

The release of extracellular vesicles has been reported to be a common feature shared by microorganisms living in marine ecosystems, as is the case for *Prochlorococcus* and *Synechococcus* (56, 57). Potential functions have been suggested for these vesicles (56, 58). Picocyanobacterial vesicles can be involved in carbon transfer, although the presence of proteins and nucleic acids within them would imply a potential role as nitrogen and phosphorus sources as well (59). Among those ORFs labeled as related to secretion processes (Table S4), 11 showed a significant downregulation, while 25 showed a significant upregulation. It is worth noting the identification of three different ORFs encoding YkuD domain-containing proteins (60) showing similar values of expression under nitrogen starvation and nanomolar nitrate concentration: 3.41 versus 3.68 (*SynWH7803_0567*), 2.13 versus 2.11 (*SynWH7803_0607*), and 2.57 versus 2.68 (*SynWH7803_0745*). YkuD is an l,d-transpeptidase catalytic domain involved in the peptidoglycan cross-linking in the cell wall, thus participating in the bacterial secretion process (58). Furthermore, a lipocalin-like domain-containing protein (*SynWH7803_0924*) has also been identified showing a strong upregulation under both conditions: 6.75 versus 6.84. The lipocalin protein family includes a large group of eight-stranded β-barrel extracellular proteins which are characterized by their ability to enclose an internal ligand-binding site for a range of small hydrophobic molecules and to form complexes with soluble macromolecules (61, 62). The upregulation of those different mechanisms of secretion in *Synechococcus* WH7803 under both nitrogen starvation and nanomolar nitrate concentrations and their involvement in the release of extracellular vesicles in this strain could be considered for future studies.

### Quantitative analysis of *Synechococcus* WH7803 proteome.

Absolute quantification was obtained using the Hi^3^ method in Progenesis QI (Waters Corporation) for 326 proteins, which corresponds to 13% of the predicted proteome. The most abundant proteins in the proteome of *Synechococcus* WH7803 were several isoforms of C-PE (UniProtKB A5GJ03, A5GJ04, Q08086, and A08087), followed by several oxidative stress response proteins such as superoxide dismutase (UniProtKB A5GMK3), ferredoxin (UniProtKB A5GN90), thioredoxin (UniProtKB A5GM53), and peroxiredoxin (UniProtKB A5GKS9). The nitrogen regulatory protein P_II_ (UniProtKB A5GNE6) is also one of the most abundant proteins in the cell, independently of the source of nitrogen used (Table S5).

**FIG 4:**
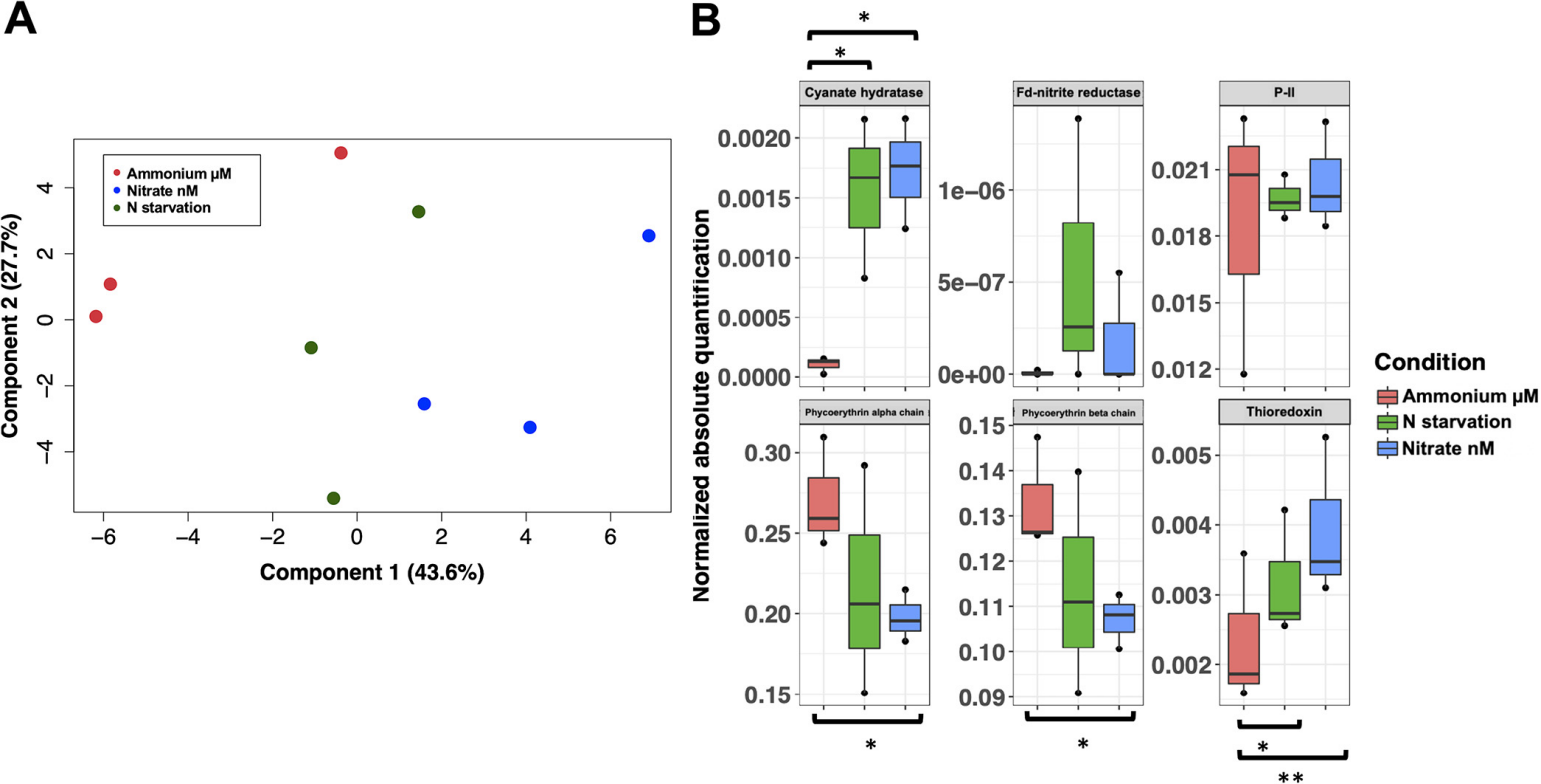
Analysis of quantitative proteomic results. (A) PCA of quantitative data from significantly changed proteins. (B) Absolute quantification from key proteins related to nitrogen metabolism and redox regulation. Ammonium μM: 800 μM ammonium; N starvation: nitrogen starvation; Nitrate nM: 800 nM nitrate. The statistically significant changes are highlighted. *, *P* < 0.05; **, *P* < 0.01.

Changes identified in the proteome were not dramatic: a total of 28 proteins changed significantly comparing nitrogen starvation versus control conditions, and 41 changed significantly comparing a nanomolar concentration of nitrate versus control conditions. Principal-component analysis (PCA) of significantly changed proteins in a nanomolar concentration versus the control (Fig. 4A) shows a slight separation between control replicates and nitrate and nitrogen starvation, supporting the hypothesis that *Synechococcus* cells sense nanomolar concentrations of nitrate and develop a specific response different from that under nitrogen starvation.

As shown by the RNA-seq data, one of the proteins with the highest increase is ferredoxin-nitrite reductase; however, protein quantification was very variable between replicates, and statistical test results were not significant (Fig. 4B). In good agreement with the results obtained in the transcriptome study, the absence of ammonium in the media promoted an increase in the abundance of the enzyme cyanate hydratase (UniProtKB A5GPQ6). This enzyme shows a 16-fold increase in 800 nM nitrate (*P* = 0.01) and a 14.8-fold increase under nitrogen starvation (*P* = 0.02).

Results of the quantification of PE (Fig. 4B) are comparable to those obtained by the total PE quantification method (Fig. 2B). PE isoforms decreased in all experimental growing conditions compared to the control; however, this change is only significant for PE class II alpha and beta chains when cultures grow in 800 nM nitrate (*P* = 0.04 and *P* = 0.03, respectively).

Nitrogen limitation induces a clear effect on the redox status of picocyanobacteria, as has been reported for *Prochlorococcus* sp. strain SS120 (63). Redox modifications and subsequent redox regulation of proteins have been linked to different processes, such as photosynthesis, transcription, translation, and protein folding (64). Thioredoxin is a small protein involved in the disulfide exchange on cysteine residues of proteins which shows a wide variety of targets in cyanobacteria (63, 65). The induction of thioredoxin A in *Synechococcus* WH7803 cells growing under starvation of ammonium is in good agreement with that general response to nitrogen limitation in cyanobacteria. Two different isoforms of thioredoxin were identified in our study (UniProtKB A5GN01 and UniProtKB A5GM53). The thioredoxin with the code UniProtKB A5GN01 showed a significant increase under nitrogen starvation and in nanomolar concentrations of nitrate (Fig. 4B).

With respect to other proteins involved in nitrogen assimilation, the sensor-transducer protein P_II_ was the only one identified in this analysis, with no significant differences between cultures growing in ammonium and those in nitrogen starvation and nanomolar concentrations of nitrate.

### Time-course effect of different nitrogen sources on nitrogen-related genes.

**FIG 5:**
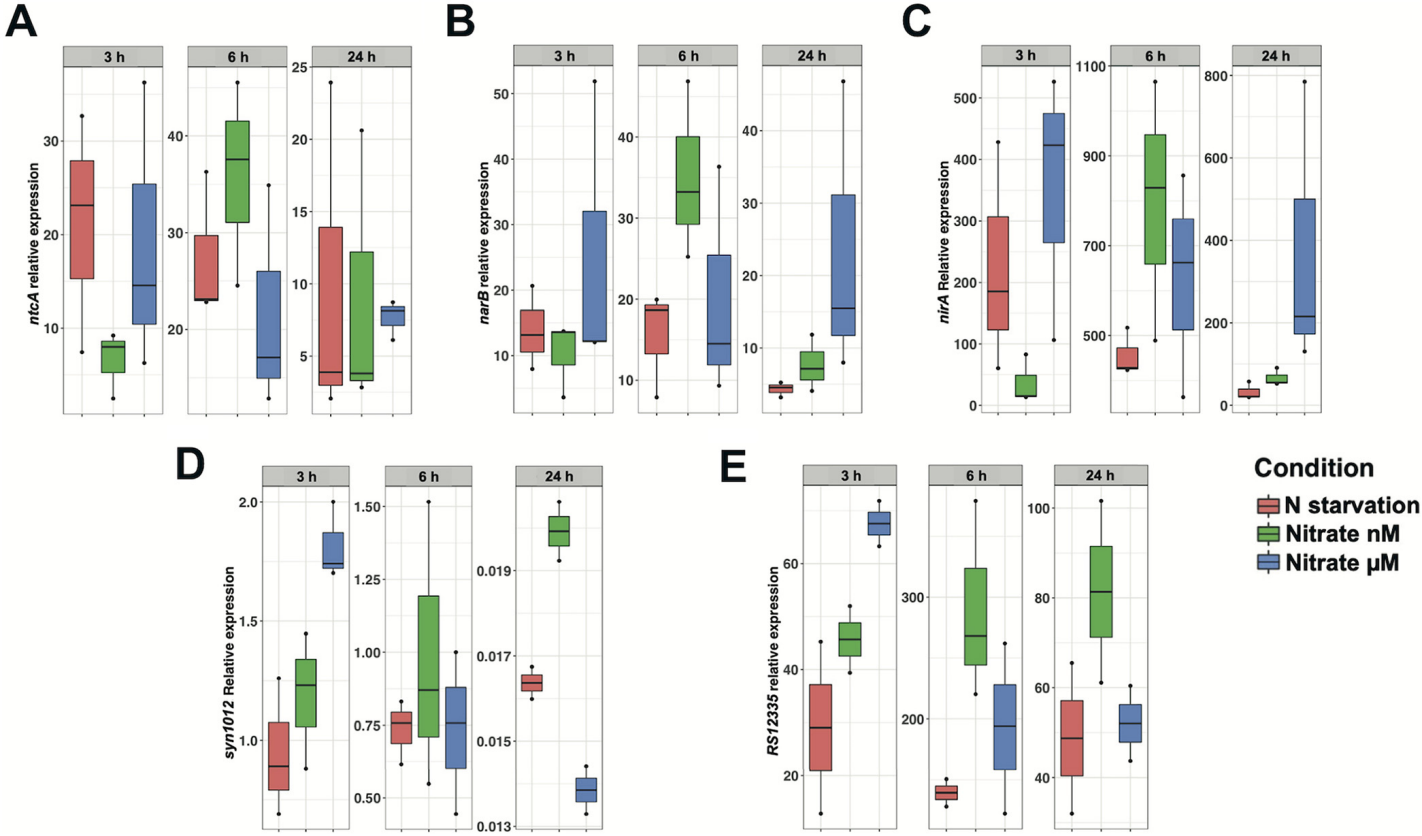
(A to E) Effect of nitrogen sources on *ntcA* (A), *narB* (B), *nirA* (C), *Syn1012* (D), and *RS12335* (E) expression. Gene expression was measured by quantitative real-time (qRT-PCR). Data are the average of three independent biological replicates.

To broaden the analysis of the response at 24 h by transcriptomic and proteomic approaches, the effect of nitrogen source at different times (3, 6, and 24 h) was also studied in *Synechococcus* WH7803 by reverse transcription-quantitative PCR (qRT-PCR) (Fig. 5). The results are represented as relative expression to the housekeeping gene *rnpB.* At 6 h, the expression of the transcriptional regulator, *ntcA*, and that of the genes encoding nitrate reductase (*narB*) and nitrite reductase (*nirA*) increased compared with N starvation. In cyanobacteria, the transcriptional factor NtcA regulates both genes encoding the enzymes involved in nitrate assimilation, nitrate reductase, and nitrite reductase, as well as *ntcA*, which also presents a promoter with an NtcA-binding site. At 24 h, the expression of *ntcA* seemed to be similar under all tested conditions. However, *narB* and *nirA* still showed higher expression values under 800 nM nitrate than in nitrogen starvation, and interestingly, the expression was even more increased with 800 μM nitrate.

Two ORFs showing a clear differential response to the absence of nitrate versus the addition of a nanomolar concentration of nitrate were selected among the nonannotated ORFs for gene expression analysis by qRT-PCR on the basis of the transcriptomic results. *SynWH7803_1012* (annotated as “hypothetical protein”) is a downregulated ORF (−7.46 versus −4.29), and *SynWH7803_RSS12335* (a small ORF placed between *nirA* and *nitM* in the *Synechococcus* WH7803 genome) is upregulated (+193.92 versus +238.82).

*SynWH7803_1012* encodes a 112-amino acid (aa) membrane protein with an extracellular N-terminal domain (residues 1 to 27), a transmembrane domain (residues 28 to 43), and an intracellular C-terminal domain (residues 44 to 112), as predicted by the PSIPRED (66) and MEMSAT-SVM (67) software. Intriguingly, a BLASTP search of its sequence against the protein sequences inferred from the *Synechococcus* WH7803 genome pointed to NtcA as one of the proteins with higher similarities. More specifically, the intracellular domain of SynWH7803_1012 showed some similarities to the DNA-binding domain of NtcA (44% identity; 60% of positives).

*SynWH7803_RSS12335* is an ORF of 135 bp which would encode a 44 aa-peptide. While PSIPRED predicted two alpha-helix segments for this peptide (from residue 5 to 17, and from residue 34 to 43), UniProt indicated 52.6% identity (65.85% of positives) with nitrite reductase from *Synechococcus* sp. CB0101 and 44.4% identity (55.6% of positives) with nitrite reductase from *Prochlorococcus* sp. NATL2A, although this peptide is clearly smaller than those enzymes. The possibility of cotranscription of *nirA*, *SynWH7803_RSS12335*, and *nitM* was tested by reverse transcription-PCR (RT-PCR) performed with RNA isolated from *Synechococcus* WH7803 cells grown with ammonium and then transferred to a medium supplied with 800 nM nitrate for 24 h. After reverse-transcription with a primer annealing just a few nucleotides from the end of *nitM*, amplification products were obtained corresponding to parts of these three genes: *nirA*, *SynWH7803_RSS12335*, and *nitM* (Fig. S4). The results indicated that cotranscription of these genes takes place.

Expression of *SynWH7803_1012* and *SynWH7803_RSS12335* was also studied in *Synechococcus* WH7803 cultures with different nitrogen sources at different times (3, 6, and 24 h) by qRT-PCR. For *SynWH7803_1012* we observed nonsignificant changes at early times (3 and 6 h) for nitrogen starvation and micromolar and nanomolar concentrations of nitrate with respect to ammonium-supplied cultures, while at 24 h, a striking downregulation of this ORF was observed under those conditions: −61.09 (N starvation), −50.18 (800 nM nitrate) and −72.19 (800 μm nitrate). Although qRT-PCR confirmed the lower downregulation of this ORF under the nanomolar nitrate conditions than under nitrogen starvation, an even higher decrease of its expression was observed after 24 h in a micromolar nitrate concentration. The function of this predicted membrane protein that was strongly downregulated after 24 h without ammonium still needs to be elucidated. The *SynWH7803_RSS12335* ORF showed a strong upregulation at each time included in this study for the three conditions, reaching the highest value at 6 h. Cultures growing with nitrate as the sole nitrogen source, at either micromolar or nanomolar concentrations, always showed a stronger upregulation than that for nitrogen starvation, and it is worth noting that the expression of this ORF was higher with the nanomolar nitrate concentration than with the micromolar concentration at 6 and 24 h. Despite being transcribed in the same mRNA and sharing a general upregulation at each time and experimental condition studied, *nirA* and *SynWH7803_RSS12335* showed a differential gene expression time course, pointing to a specific RNA processing.

## DISCUSSION

Nitrogen metabolism in the marine cyanobacterium *Synechococcus* has been investigated to a far lesser extent than in freshwater cyanobacteria, and while there are similarities in some strains, there are also some intriguing differences (37, 41, 68, 69). In this work, the capacity of *Synechococcus* to cooccur with *Prochlorococcus* has been assessed despite the apparently lower capability of marine *Synechococcus* to take ammonium as a nitrogen source, even though it has a higher cell nitrogen quota (41). For that reason, this study focused on nitrate, the main oxidized form of nitrogen that many *Prochlorococcus* strains isolated to date are not able to assimilate (42, 70, 71). Strains from marine *Synechococcus* and *Prochlorococcus* cohabit at the upper surface layer of the water column where the concentrations of the oxidized forms of nitrogen are low (2). The ability to transport and metabolize nitrate at nanomolar concentrations might be a key feature which contributes to *Synechococcus* strains thriving in those oceanic areas where nitrogen availability is limiting for *Prochlorococcus* strains.

The genomic context study showed the differences in the arrangement of the nitrogen assimilation pathway between the marine and the freshwater cyanobacteria. Although those genes seem to be regulated by NtcA in both cases (Fig. 1), we expected differences in the response when *Synechococcus* WH7803 is subjected to different nitrogen sources to grow. The cultures are routinely maintained in a medium containing ammonium. The growth curve showed that under 800 μM ammonium or nitrate, the rate of growth is higher than that under nitrogen starvation, as was expected (Fig. 2A). However, when cells are in a medium containing 800 nM nitrate, the growth remains intermediate. Interestingly, our results showed that after 24 h of nitrogen starvation, the reduction of the PE content was about 16%, in good agreement with the results from Kana and coworkers that showed minor loss of PE, 25%, after 3 days of nitrogen starvation in *Synechococcus* WH7803 (from mesotrophic regions) compared with WH8018 (coastal strain), which lost 85% of the pigments (48), the most common response. That unusual response from *Synechococcus* WH7803 may reflect an adaptation to surface waters with a limited nutrient availability. Furthermore, the reduction of the PE content correlated with the decrease in the expression of the genes encoding the key enzymes involved in the synthesis of this pigment, as was the case for the genes encoding the subunits from photosystems I and II.

The ferredoxin-nitrate reductase catalyzes the reduction of nitrate to nitrite, which is the first step in the assimilatory reduction of nitrate (72, 73). This key enzyme in the nitrogen metabolism of *Synechococcus* WH7803 was further studied. Our results showed nitrate reductase activity even when the cells were grown under nitrogen deprivation, indicating that nitrate was not required as an obligate inducer. However, when the cells grew with nitrate, the activity was higher than that detected after 24 h under nitrogen starvation, as shown for Anacystis nidulans and *Nostoc* sp. strain PCC 6719 (74). Ammonium represses the nitrate reductase in freshwater cyanobacteria (73–75). The ammonium does not exert its effect directly, but it has to be incorporated in carbonated skeletons through the GS-GOGAT pathway (74). In *Synechocystis* sp. PCC 6803, it has been described that ammonium repressed *nirA* and *narB* expression (76). Our findings showed a different response in *Synechococcus* WH7803, since nitrate reductase activity was found even when the cells were growing on ammonium, in agreement with the results of Zehr and coworkers (77), where they strongly suggest that phytoplankton contain a constitutive nitrate reductase activity under ammonium that can be used to rapidly reduce nitrate and allow its utilization when it is available.

It has been described that the nitrate assimilatory genes are finely regulated in freshwater cyanobacteria (22, 27). 2-OG, the major intracellular signal signaling the C/N balance, participates in this regulation. When nitrogen is limiting growth, accumulation of 2-OG activates the transcription factor NtcA to induce the transcription of the nitrate assimilation genes (22, 25, 27, 78). For the first time, we quantified the changes in the intracellular level of 2-OG in the marine cyanobacterium *Synechococcus* WH7803 (Fig. 2D). Our results confirmed that 2-OG increased under nitrogen starvation, upregulating the expression of *ntcA* and other nitrogen-related genes controlled by NtcA, such as *ntcA* itself, and *narB*, *nirA*, *glnA*, or *glnB*, which exhibit putative NtcA-regulated promoters in *Synechococcus* WH7803. As a result of this upregulation, an increase in the glutamine synthetase activity was also observed, as well as a larger amount of the sensor-transducer protein P_II_.

However, *Synechococcus* WH7803 cells show many other responses which clearly differ from those described for freshwater cyanobacteria. For instance, NtcA-regulated genes such as *icd* or *pipX*, increasing their transcriptional activity under nitrogen starvation in freshwater strains, neither exhibit putative NtcA-binding sites in their promoter, nor show an upregulated expression in *Synechococcus* WH7803. An additional unusual feature is the detection of the nitrate reductase activity in cultures growing with ammonium as the sole nitrogen source, since *narB* is an NtcA-regulated gene not usually expressed when ammonium is available and there is a low intracellular concentration of 2-OG.

The responsiveness of *Synechococcus* WH7803 to nanomolar concentrations of nitrate has been assessed in this work, since that is the range of nitrate concentration found in wide areas of the ocean where marine *Synechococcus* thrives. The results of this study show some examples of a differential response to nanomolar nitrate concentrations with respect to nitrogen starvation, indicating that *Synechococcus* WH7803 can perceive and adjust its metabolic pathways to this condition. It has been observed how PE content varies between the absence of nitrogen sources and the addition of nitrate at a nanomolar concentration. In agreement with these results, the expression of genes encoding the enzymes involved in PE synthesis showed a less severe decrease when a low quantity of nitrate was available, and that same response was also observed for genes encoding the photosystem I and II subunits. Curiously, genes involved in nitrogen assimilation were those which exhibit almost equal values of expression in both conditions, showing a differential response only at early times, as was the case for the expression of *ntcA*, *narB*, and *nirA* when determined at 6 h by qRT-PCR. These results differentiating nitrogen starvation from nanomolar nitrate concentrations obtained at early times could indicate that after 24 h the added amount of nitrate has been nearly consumed, and the *Synechococcus* WH7803 cells are starting to adapt to the absence of nitrogen sources.

The specific response of genes not related to known functions, *SynWH7803_1012* and *SynWH7803_RSS12335* (which are down- and upregulated, respectively, when comparing cultures grown in nanomolar nitrate versus no nitrogen) is intriguing and deserves further research. It is particularly surprising that SynWH7803_1012 and SynWH7803_RSS12335 show significant homology with some parts of NtcA and nitrite reductase, respectively. This might suggest that these ORFs could have evolved from previously existing genes involved in standard nitrogen assimilatory pathways, which were adapted to respond to very low nitrate concentrations in marine environments.

Another remarkable aspect of our results is the identification of P_II_ as one of the most abundant proteins in *Synechococcus* WH7803. To our knowledge, the main reported function of P_II_ thus far is the control of the C/N balance in cyanobacteria (79, 80). However, there is increasing evidence showing that P_II_ is also involved in the control of transporters, signaling molecules (c-di-GMP), and cofactors (NAD^+^) (81). Although P_II_ is the most abundant protein among nitrogen regulators in the freshwater model Synechococcus elongatus PCC 7942 (23), regulatory proteins usually appear in low relative abundance (80), so this large amount of P_II_ inside *Synechococcus* WH7803 cells would point out to possible additional, and yet unknown, functions for this protein.

Primary production in the oceans is supported by two major forms of nitrogen: ammonium, which is a reduced form provided by the recent decomposition of organic matter in the water column, and nitrate, which is an oxidized form and diffuses into the euphotic zone from deep water. It is generally believed that ammonium is a major source of nitrogen for phytoplankton in oligotrophic oceans. However, some studies suggested that nitrate might also play an important role (40, 77). This importance is reflected by mixing events at the ocean water that promote changes in the concentration of nitrate. These mechanisms require that phytoplankton, which may have been growing primarily on ammonium, become rapidly capable of transporting, reducing, and incorporating nitrate (77). Moreover, studies focused on the effect of different nitrogen sources on the enzymes and regulatory proteins involved in these pathways usually do not consider the scarce concentrations at which either nitrate or ammonium could be available in some oceanic areas. To our knowledge, this is the first integrated transcriptomic and proteomic study focused on nitrogen assimilation in a marine picocyanobacterium revealing that very low concentrations of nitrate are actually perceived by marine *Synechococcus*, leading to specific changes in its transcriptome and proteome, which are different from those provoked by nitrogen starvation. Further studies are required to analyze whether this organism has developed uptake mechanisms allowing it to scavenge nanomolar concentrations of nitrate (as suggested by preliminary studies from our team) and the molecular underpinnings enabling its detection.

## MATERIALS AND METHODS

### *Synechococcus* strain and culture conditions.

*Synechococcus* WH7803 was grown in a chemically defined artificial seawater medium (ASW) (82). Cells were grown in polycarbonate Nalgene flasks in a culture room set at 24°C under continuous blue irradiance (40 μmol quanta m^−2^ s^−1^). Growth was determined by measuring the absorbance of cultures at 750 nm, and cells were collected during the exponential phase of growth, when absorbance at the indicated wavelength was 0.1.

### Experiments and preparation of cell extracts.

To prepare the experiments, cultures with 800 μM ammonium (8 L) at 0.1 units of absorbance at 550 nm were centrifuged at 26,000 × *g* for 8 min at 24°C using an Avanti J-25 Beckman centrifuge equipped with a JA-14 rotor. Cell pellets were washed twice with ASW medium with no nitrogen source added and then resuspended in ASW medium supplemented with either no nitrogen or different forms of nitrogen sources, namely, ammonium [(400 μM NH_4_]_2_SO_4_), and nitrate (800 nM or 800 μM KNO_3_). Cultures were then kept under standard light and temperature conditions for 24 h. Cells were harvested by centrifugation at 26,000 × *g* for 8 min at 4°C. Cell pellets were resuspended in 2 mL of 25 mM ammonium bicarbonate for proteomic studies, in 10 mM sodium acetate (pH 4.5), 200 mM sucrose, and 5 mM EDTA for RNA analysis, and in 50 mM Tris-HCl, (pH 7.5), for protein assays. Samples for proteomic studies and protein assays were stored at −20°C until use and those for RNA analysis, at −80°C.

### Quantification of total protein concentration.

Protein concentration in soluble fractions was determined using the Bio-Rad protein assay according to the Bradford method (83).

### Determination of quantum yield of photosystem II.

A total of 250 mL of culture was centrifuged at 26,000 × *g*, for 8 min at 4°C. The cell pellets were resuspended in 2 mL of ASW medium and used to fill a 24-well culture plate (Biofil). The samples were incubated in the dark for 30 min. Then, the fluorescence of the chlorophyll was measured using a PAM WALZ IMAG-K5 imaging system. The photosynthetic radiation used was 36 μmol quanta m^−2^ s^−1^.

### Determination of PE.

Phycoerythrin (PE) was measured as described by Wyman (84). PE concentration was determined by measuring absorption at 542 nm with a molar extinction coefficient for the hexamer of 2.15 × 10^6^ M^−1 ^cm^−1^.

### Determination of intracellular concentration of 2-OG.

After the samples were thawed on ice, the extracts were centrifuged for 10 min at 16,900 × *g* and 4°C. The supernatants were used for the determination of 2-OG using an enzymatic method based on the oxidation of NADPH in the reaction catalyzed by the glutamate dehydrogenase as previously described (85). The NADPH consumption during the reaction was monitored by measuring the absorbance at 340 nm for 10 min at 35°C.

### Determination of enzymatic activities.

The glutamine synthetase transferase (85, 86) and the nitrate reductase (87) activities were determined as previously described.

### RNA isolation and quantitation of gene expression by qRT-PCR.

Total RNA from 500-mL *Synechococcus* culture samples was isolated using the TRIsure RNA isolation reagent (Bioline) as previously described (39). The synthesis of cDNA from RNA samples was carried out using the iScript cDNA synthesis kit (Bio-Rad) as recommended by the manufacturer. Specific primers were designed using the software Primer3Plus (http://primer3plus.com) on the base of the published genome of the *Synechococcus* WH7803 strain. The specificity of the qRT-PCRs was checked for single amplification of DNA fragments of the expected size by agarose gel electrophoresis and for identification by Sanger sequencing (carried out at the genomic facilities of Servicio Centralizado de Apoyo a la Investigación (SCAI); Universidad de Córdoba). qRT-PCRs were performed as previously described (39) by using the *rnpB* expression as the internal control for quantitation. Gene expression determinations were carried out as described (85), according to the Pfaffl method (88).

### RNA sequencing and transcriptomics data analysis.

Total RNA extraction for RNA sequencing was performed as described for qRT-PCR, including an ammonium acetate precipitation step. RNA samples treated with RNase-free DNase I (Ambion) were purified with the RNA Clean & Concentrator-5 kit (Zymo Research). The purity and concentration of each total RNA sample were measured using a NanoDrop ND-1000 spectrophotometer (Thermo Fisher Scientific), while their quality and integrity were assessed using a Bioanalyzer 2100 instrument (Agilent Technologies) to obtain the corresponding electropherograms. Libraries for Illumina sequencing were prepared using ribosome-depleted RNA samples. RNA-sequencing performed on the Illumina HiSeq 2000 platform and alignment procedures were conducted by Sistemas Genómicos (Valencia, Spain).

Raw reads were mapped to the *Synechococcus* WH7803 genome (ID NC_009481.1), and the quality of these reads was controlled using Picard Tools (http://broadinstitute.github.io/picard/). The assembly and identification of the transcripts were performed using Cufflinks v2.11 software, while the transcript quantification was carried out with the HTSeq algorithm (http://www-huber.embl.de/HTSeq/doc/overview.html). To assess the normalization of the differential expression analysis, the DESeq2 algorithm was used applying a negative binomial distribution model (89). The whole-transcriptomics data analysis was conducted by Sistemas Genómicos (Valencia, Spain).

Three biological replicate experiments of *Synechococcus* WH7803 were prepared, and each one was split into three growing conditions: 800 μM ammonium (control), 800 nM nitrate, and nitrogen starvation. After 24 h, RNA was isolated as described in Materials and Methods; the quality parameters of the isolated RNA are shown in Table S6. The total qualified mRNA-based sequence reads identified after a step data filtering process are in Table S7. Regarding the quality of the sample, the percentage of the properly pair indicates the high quality of the mapping. Moreover, the data reflect homogeneity among triplicates. In order to get a better comparison among samples, these have been normalized with respect to the length of the genes and the size of the library, as shown in Fig. S5A and B The study of the quality of the differential expression among the samples (Fig. S5C and E) showed that the obtained results fit the model. Furthermore, the MA plot (M is the log ratio and A is the mean average) (Fig S5D and F) shows the number of genes that are differentially expressed between conditions.

### Quantitative proteome determination by liquid chromatography-tandem mass spectrometry analysis.

Three biological replicates were processed for the proteomic analysis as previously described, using trypsin as the protease (90, 91). The peptides generated were separated on an Ultimate 3000 Dionex ultra-high-pressure liquid chromatography (UHPLC) instrument (Thermo Fisher Scientific) using the following chromatographic conditions: preconcentration in a C_18_ precolumn (Acclaim PepMap100, 5 μm, 0.3 mm by 5 mm, Thermo Fisher Scientific) for 3 min at 5 μL/min in 98% acetonitrile (ACN)/0.1% trifluoroacetic acid (TFA) as the loading buffer. Then, a chromatographic gradient of 4 to 40% ACN was performed on a C_18_ nano-column (Acclaim PepMap rapid-separation liquid chromatography [RSLC] instrument, 75 μm by 50 cm, Thermo Fisher Scientific) at a flow rate of 300 nL/min for 60 min. The total chromatography time was 85 min. Eluting peptides were analyzed on an Orbitrap Fusion device (Thermo Fisher Scientific) operated in top 50 data-dependent acquisition mode, selecting for fragmentation the top 50 ions of the highest intensity from each full scan at a maximum duty cycle time of 3 s. The mass range acquisition in full-scan mode was 400 to 1,500 *m/z* in the Orbitrap at a resolution of 120 K, a 4 × 10^5^ automatic gain control (AGC) ion count target, and a maximum injection time of 50 ms. Tandem mass spectrometry (MS^2^) was performed by quadrupole isolation at 1.2 Th. Only precursors with a charge state of 2 to 5 were sampled for MS^2^, setting a dynamic exclusion window of 15 s and allowing a 10-ppm tolerance for the selected precursor. Fragmentation was performed by collision-induced dissociation (CID) at a normalized collision energy of 35. Fragment ions were analyzed in the ion trap. Liquid chromatography-tandem mass spectrometry analysis was carried out at the proteomic facilities of SCAI.

Absolute quantification of proteins was calculated using the Hi^3^ strategy in Progenesis QI (Waters Corporation). “Normalize to all proteins” was selected in Progenesis QI as the normalization method to correct from protein loading. The peak list generated in Progenesis QI was searched against a database containing all entries for *Synechococcus* WH7803 in UniProt (www.uniprot.org) (retrieved on 8 May 2019; containing 2,530 entries). As the internal standard for absolute quantification, rabbit phosphorylase B (UniProtKB P00489, Waters Corporation) was added to each sample (50 fmol on column). Search parameters were set as follows: trypsin was selected as the protease for digestion, allowing one missed cleavage and setting carbamidomethylation of cysteines as fixed modification and methionine oxidation as variable modification. Precursor mass tolerance was set at 10 ppm, product ion tolerance at 0.5 Da, and false-discovery rate (FDR) at 1%. Since nonaxenic cultures were used in the experiments, protein concentration was further corrected using total protein abundance to account for differences in the populations of *Synechococcus* in cultures. For a protein to be considered significantly differentially expressed, we used the following criteria: identified and quantified using at least two unique peptides and with a *P* value of ≤0.05.

### Data availability.

The proteome data set is available at the PRIDE repository with the following information: project name: “Marine Synechococcus sp. strain WH7803 shows specific adaptative responses to assimilate nanomolar concentrations of nitrate”; project accession no.: PXD031142; project DOI:10.6019/PXD031142. Transcriptome data have been deposited in the NCBI public repository at https://www.ncbi.nlm.nih.gov/bioproject/PRJNA830281.
