# Supplementary material for: Marine Synechococcus sp. Strain WH7803 Shows Specific Adaptative Responses to Assimilate Nanomolar Concentrations of Nitrate
Source: Microbiol Spectr. 2022 Jul 19;10(4):e00187-22. doi: 10.1128/spectrum.00187-22 (PMC9430850; doi:10.1128/spectrum.00187-22)
Supplement: Supplemental file 1 — Tables S1 and S2, captions for Tables S3 to S7, and Fig. S1 to S5. Download spectrum.00187-22-s0001.pdf, PDF file, 0.9 MB [file spectrum.00187-22-s0001.pdf]

Supplementary Information

Supplementary Table 1: Genome discovery via RNA seq study. List of the total identified genes, gene description, gene ontology and Interpro information.

Supplementary Table 2: Differential gene expression comparing control and nitrogen starvation condition. Cut-off > 1.5-fold change and pValue adjust < 0.05.

Supplementary Table 3: Differential gene expression comparing control and nanomolar of nitrate condition. Cut-off > 1.5-fold change and pValue adjust < 0.05.

Supplementary Table 4: Blast of the hypothetical proteins. Hypothetical proteins with significant changes (cut-off > 1.5-fold change and pValue < 0.05), up-regulated tab and down-regulated tab. The gene ID, the description of the gene, the description from Uniprot and the fold change are provided in each excel sheet.

Supplementary Table 5: Proteomics identification and absolute quantification.

Supplementary Table 6: RNA parameters of quality. Control: cultures grown under ammonium; N Starv: cultures grown under nitrogen starvation; Nitrate: cultures grown under 800 nM of nitrate. 1, 2 & 3 mean the three biological replicates.

# Sample	Name sample	A_{260}	$A_{260/280}$	$A_{260/230}$
**1**	Control -1	11.2	2.05	2.22
**2**	N Starv -1	10.5	2.06	2.28
**3**	Nitrate -1	10.6	2.08	2.27
**4**	Control -2	20.3	2.12	2.32
**5**	N Starv -2	17.3	2.13	2.23
**6**	Nitrate -2	18.8	2.11	2.32
**7**	Control -3	20.6	2.08	2.18
**8**	N Starv -3	17.3	2.06	2.13
**9**	Nitrate -3	18.4	2.07	2.16

Supplementary Table 7: Statistics of RNA-seq transcriptomics analysis. Control: cultures grown under ammonium. 1, 2 & 3 mean the three biological replicates.

Sample	Mapped Reads	Properly Pair	% Properly Pair
Control-1	12429138	11608044	93,39
Control-2	14126663	12899992	91,32
Control-3	15878739	14787746	93,13
Nitrogen Starvation-1	9966690	9265768	92,97
Nitrogen Starvation-2	9904925	9121534	92,08
Nitrogen Starvation-3	11660919	10817146	92,76
800 nM NO3-1	15187883	14052176	92,52
800 nM NO3-2	11359730	10387670	91,44
800 nM NO3-3	12918217	11626800	90

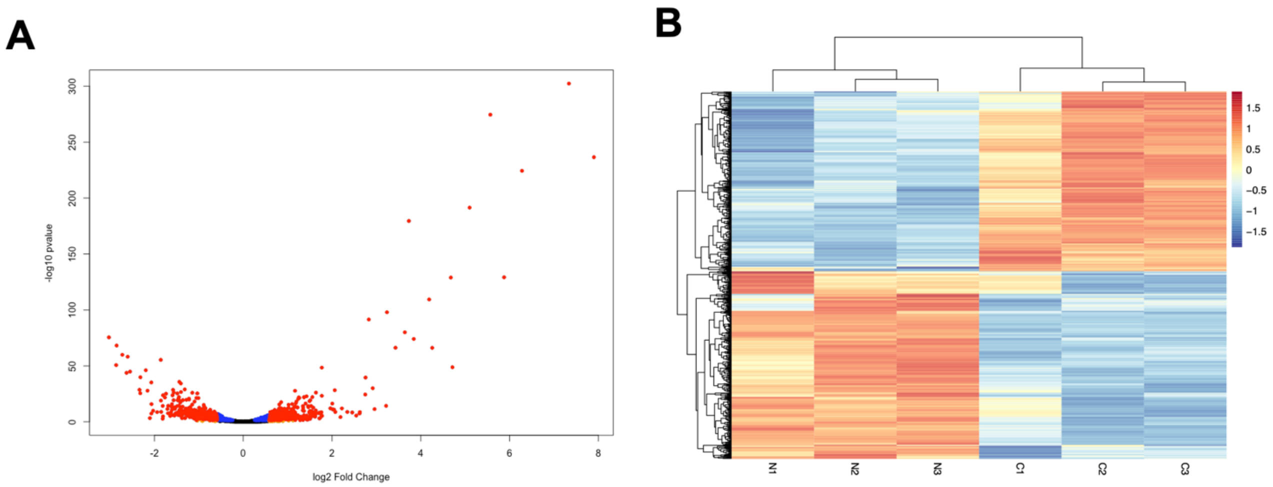

Supplementary Figure 1: Differentially expressed genes in *Synechococcus* WH7803 in the different conditions tested. A. Volcano Plot for 800 nm nitrate condition. **B.** Heatmap for the nitrogen starvation (N) comparison with control (C) conditions. 1, 2 & 3 means biological replicates.

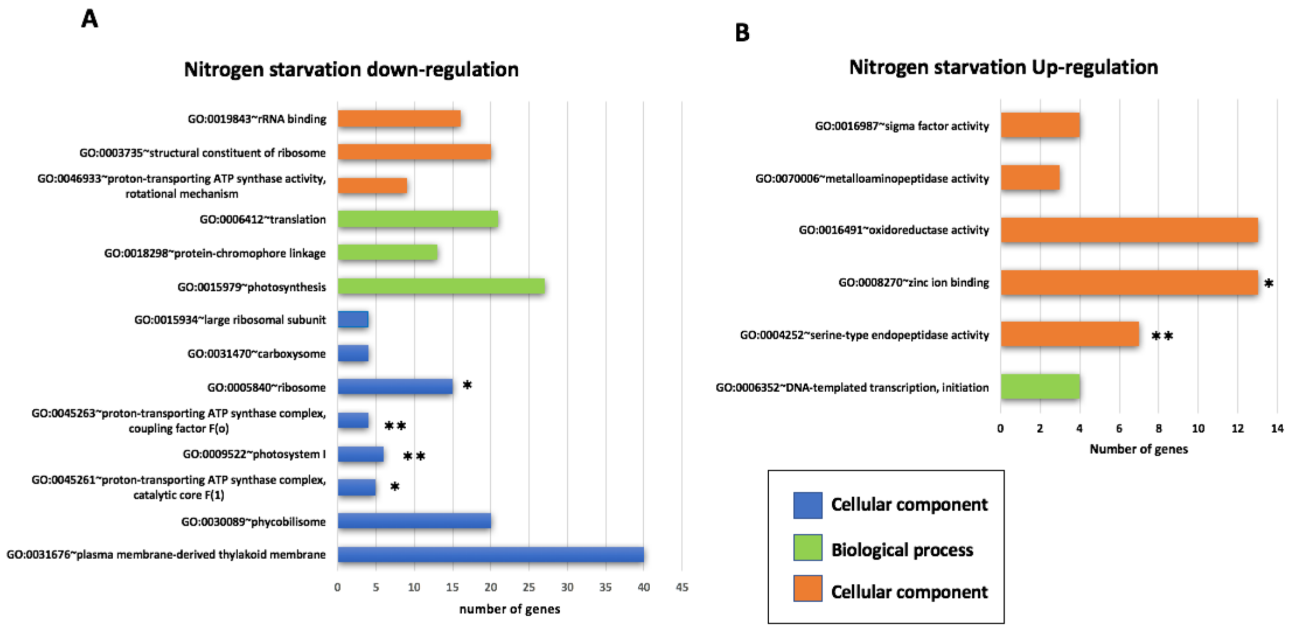

Supplementary Figure 2: DAVID Functional enrichment analysis, functional gene ontology (GO) analysis of biological process. A. Genes down-regulated under nitrogen starvation. **B.** Genes up-regulated under nitrogen starvation. Statically significant group mentioned with * (pvalue < 0.05); ** (pvalue < 0.01).

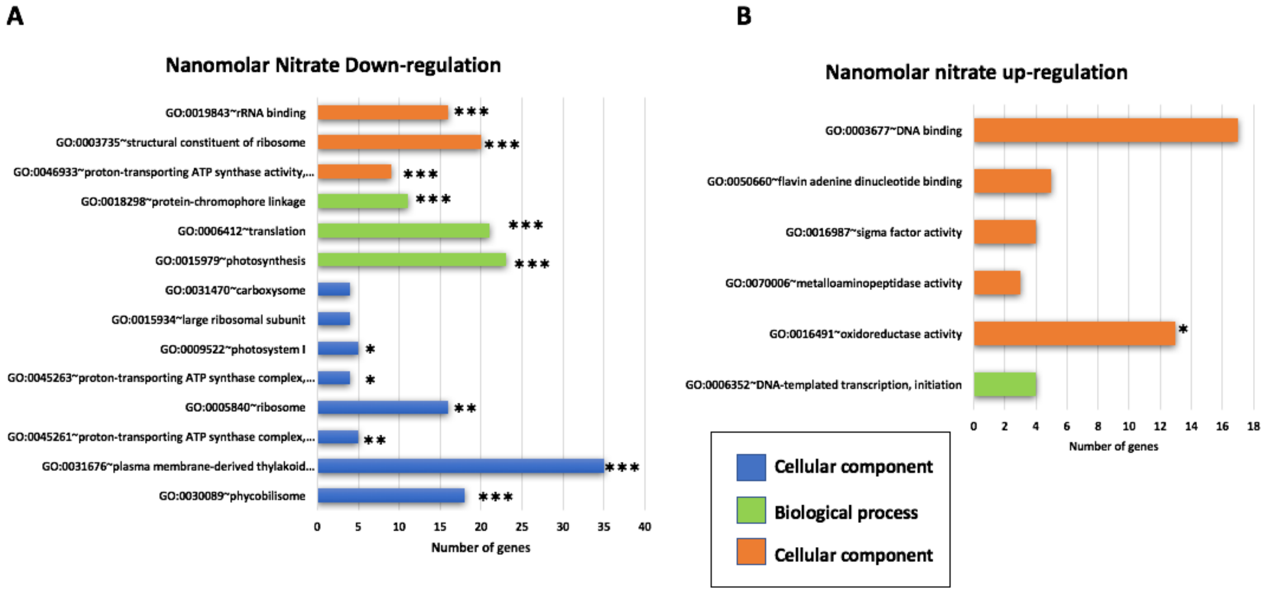

Supplementary Figure 3: DAVID Functional enrichment analysis, functional gene ontology (GO) analysis of biological process. A. Genes down-regulated under nanomolar concentration of nitrate. **B.**

Genes up-regulated under nanomolar concentration of nitrate. Statically significant group mentioned with * (pvalue < 0.05); ** (pvalue < 0.01) and *** (pvalue < 0.001).

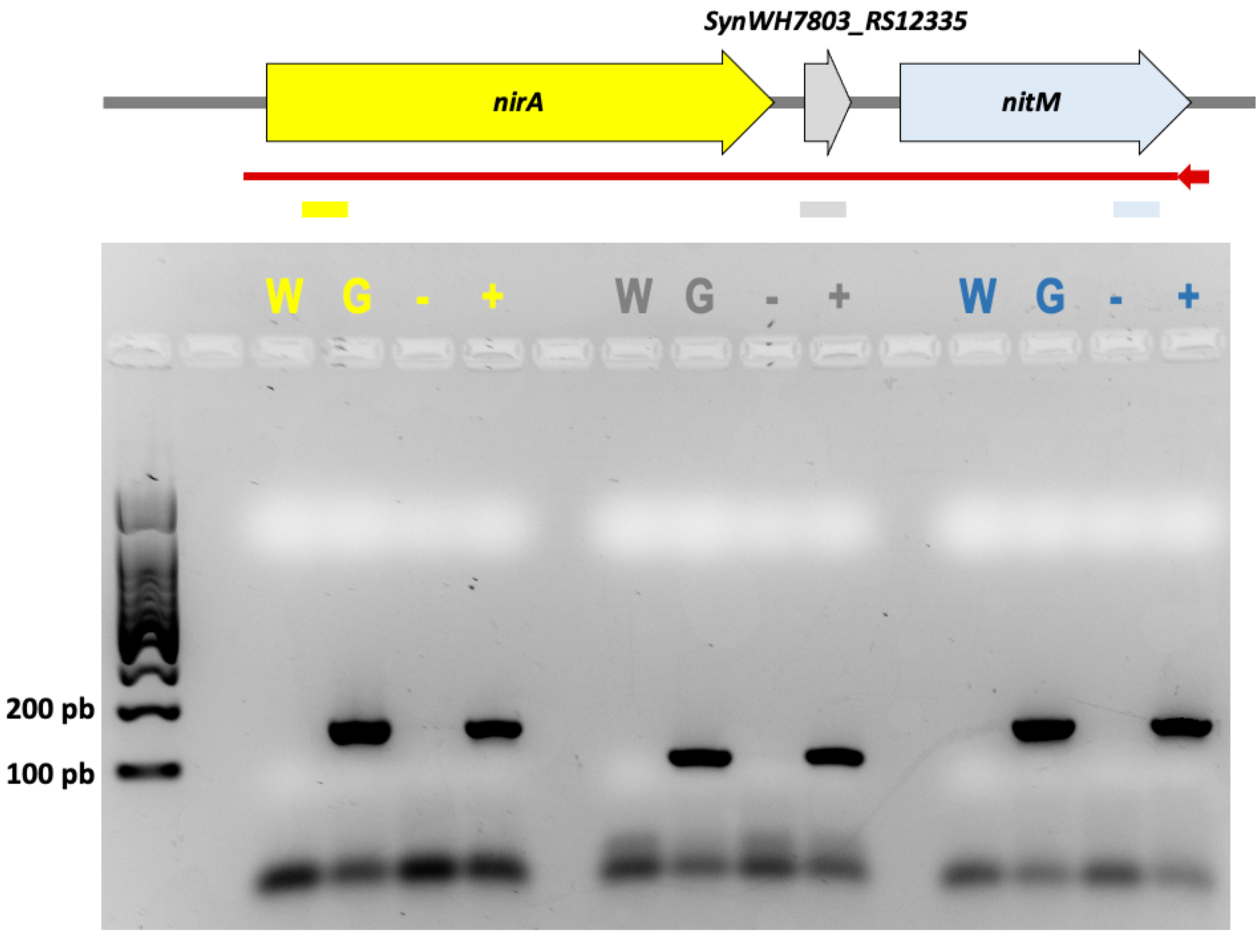

Supplementary Figure 4. RT-PCR analysis of the expression of the *nirA-SynWH7803_RS12335-nitM* gene cluster in *Synechococcus* sp. WH7803. Retrotranscription was carried out with oligonucleotide primer 7803_2493-1 (position indicated by the red arrow) and RNA isolated from cells grown in cultures with 800 µM ammonium and incubated for 24 h in the presence of 800 nM nitrate. The positions of the primers used for amplification correspond to the ends of the segments indicated in the scheme (yellow for *nirA*, gray for *SynWH7803_RS12335* and blue for *nitM*). w or g, water or total genomic DNA used as the template for amplification with the corresponding primers; + or -, RNA samples subjected or not to retrotranscription. Amplification products were only observed in those PCR reactions including either genomic DNA or cDNA.

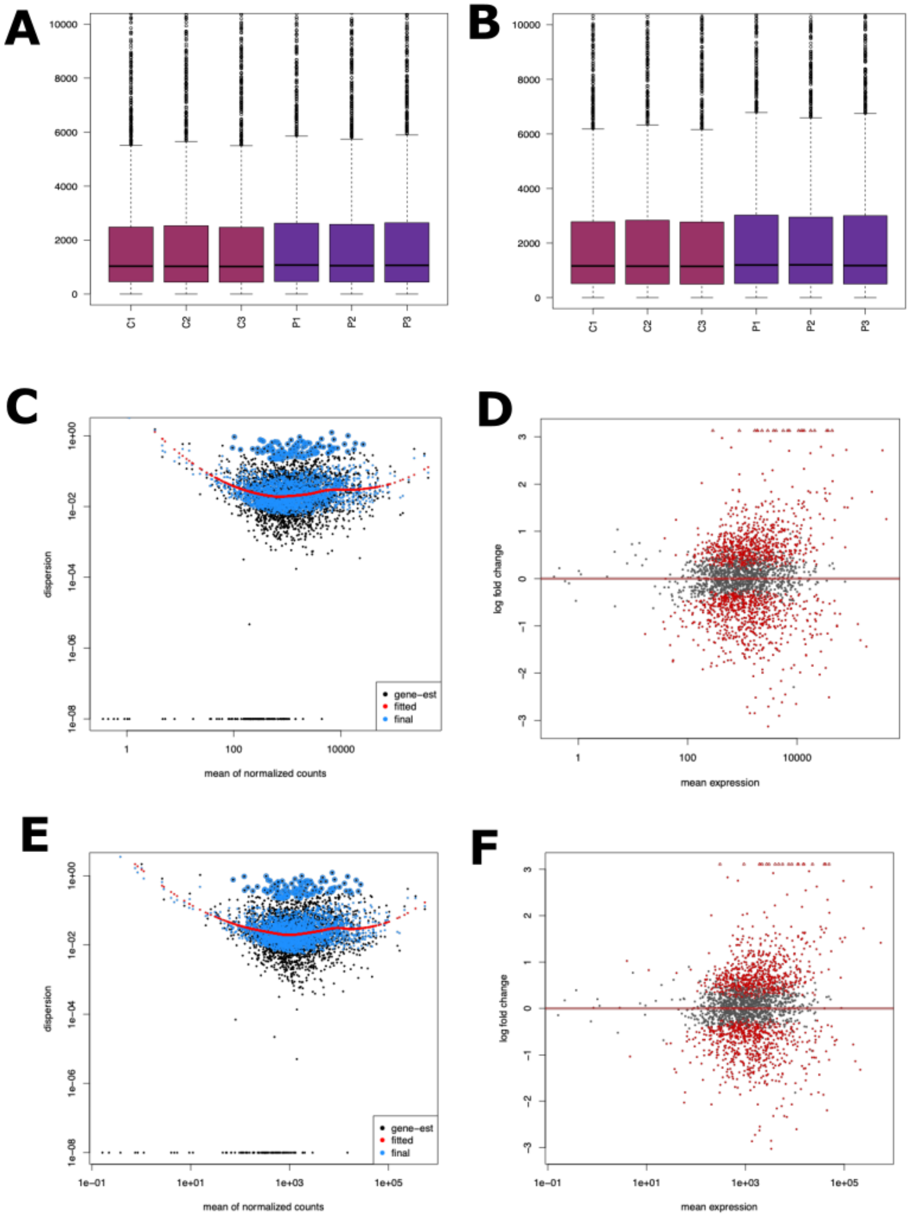

Supplementary Figure 5: Normalization of the samples. A. Nitrogen starvation (P) vs Ammonium (C). **B.** Nitrate (P) vs Ammonium (C). Dispersion of the gene expression (**C, E**) and MA plots **(D, F**). Nitrogen starvation vs Ammonium (**C, D**) and Nitrate vs Ammonium (**E, F**). C1-3 and P1-3 means three biological replicates.
